# Development of strategies to promote healthy sexuality based on Iranian girls perspective about the role of virginity in the construction of their sexuality: an explanatory sequential mixed method study protocol

**DOI:** 10.1186/s12978-021-01299-1

**Published:** 2022-02-24

**Authors:** Somayyeh Naghizadeh, Raziyeh Maasoumi, Farideh Khalajabadi-Farahani, Mojgan Mirghafourvand

**Affiliations:** 1 Department of Midwifery and Reproductive Health, School of Nursing and Midwifery, Tehran University of Medical Sciences, P.O. Box: 1419733171, Tehran, Iran; 2 Nursing and Midwifery Care Research Centre, School of Nursing and Midwifery, Tehran University of Medical Sciences, P.O. Box: 1419733171, Tehran, Iran; 3 Department of Population & Health, National Population Studies and Comprehensive Management Institute of Iran, Tehran, Iran; 4 Department of midwifery, Social Determinants of Health Research Centre, Tabriz University of Medical Sciences, Tabriz, Iran

**Keywords:** Virginity, Sexuality, Iranian girls, Healthy sexuality

## Abstract

**Background:**

The modern Iran is a blend of tradition and modernity, but its dominant culture is still traditional and takes pride in female virginity. On the other hand, the influential factors such as modernity, education, social networks, global communication, influence from advanced Western societies, have obviously led to the emergence of a freer sexual attitude associated with less significance of virginity. Since the traditional, cultural and religious significance of virginity among Iranian girls can shape their sexual behaviors, therefore, the aim of the present study is development of strategies to promote healthy sexuality based on Iranian girls perspective about the role of virginity in the construction of their sexuality.

**Methods/design:**

This is a two-stage study; an explanatory sequential mixed-methods follow-up design will be employed in the first stage, which consists of two quantitative and qualitative phases. The first phase is the cross-sectional survey that will be conducted on 700 single girls born in the 1970s, 1980s and 1990s. The sample will be selected using the cluster sampling method in the health centers of Tabriz-Iran. The determined sample size will be divided among the selected health centers based on the quota criterion, and eligible households will be selected randomly from the said centers using the SIB website (sib.tbzmed.ac.ir). To collect the required data in the quantitative phase, we will use a researcher-made questionnaire to evaluate the girls’ views about virginity and its influential factors, designed based on the theory of “social construction of sexuality”. After quantitative data collection and analysis, the findings inform qualitative data collection and analysis. The qualitative phase of the study will be conducted on girls living in Tabriz using in-depth and semi-structured individual interviews and purposive sampling method to collect the required qualitative data. The collected data will be analyzed using the conventional content analysis approach. The findings of two phase will be integrated for further explanation and interpretation to be used in the second stage. In the second phase of this study, a nominal group meeting will be held with the participation of reproductive and sexual health experts. The strategies extracted from the results of the first phase and review the texts in this meeting will be provided to experts and after receiving the opinions and ideas of the relevant group of experts and prioritizing them, appropriate strategies to improve and promote the healthy sexuality of Iranian girls will be presented.

**Discussion:**

This study is one of the few studies conducted in the field of sexual health and culture in Iran, that using a “mixed-methods” approach to determine and explain the role of virginity in the construction of the sexuality from the Iranian girls perspective. We hope that this study can present evidence-based documents from the latest physical, psychological and social developments in young Iranian girls’ sexuality and that the presented healthy sexuality promotion strategies, which will be based on Iranian socio-cultural developments, can provide the basic information required for policy-making and planning for young girls’ sexual health. It is also hoped that the findings of this study will be useful in culture-based sexuality education and support for reproductive and sexual health care for the young Iranian generation.

## Plain language summary

Custom, culture and religion in many communities impose virginity on the women of their communities. According to the traditional and conservative Iranian culture, premarital sexual relations are forbidden, female virginity is honor, and it is valuable and necessary for a girl to maintain and take care of her virginity. Attitudes toward sexual relationship and its moral aspects have changed dramatically in many parts of the world in recent decades. Iran, as a conservative society, is no exception to this rule and has experienced substantial social and attitudinal changes over the past decades. This is a two-stage study; an explanatory sequential mixed-methods follow-up design will be employed in the first stage, which consists of two quantitative and qualitative phases. At first quantitative data collection and analysis will be conducted, then the findings inform qualitative data collection and analysis. The findings of two phase will be integrated for further explanation and interpretation to be used in the second stage. In the second stage of the study, the researchers will develop appropriate strategies for the girls’ sexual health promotion by a combination of the relevant experts’ views obtained in the nominal group meeting and the results extracted from a review of literature as well as the results of the quantitative and qualitative phases of the first stage. it seems that the present study, by employing a better quantitative and qualitative approach, can clarify the young generation’s view in the socio-cultural background of Iran and explore the possible inconsistency and contradictions in the modern Iranian society to determine whether the traditional view of the concept of virginity in Iran is still dominant, or it has been changed by factors such as modernization, global communications, influence from advanced Western societies.

## Background

“Virginity” has a French root in which “Vir” means “man” and “Genere” means “created for”. The term has its roots in medieval Western culture and refers to the ownership of a man over a woman [1], and was used for a woman who has never had a sexual relationship with a man [2, 3]. However, the participants of a study conducted by Carpenter in 2002 identified penile-vaginal intercourse as the act denoting their virginity loss [4].

Virginity is a complex social concept [4, 5] with different definitions for people with different cultural backgrounds [6]. In advanced societies such as the United States, sexual activity among adolescents and youth is customary and considered the norm. In fact, changes in social and sexual norms in these societies have caused most people to have pre-marital sexual relationships (men 92.2% and women 91.9% [7]). In these societies, males are less inclined to partner with virgin females, and virgins are not considered good sexual partners [1, 8–10].

However, in traditional patriarchal societies (where gender roles are clearly defined and very rigidly executed), a woman is important not only in terms of fertility and personality, but also in terms of virginity [11]. In many countries such as Zimbabwe [12], Zambia [13], Ethiopia [14], Ghana [2], Democratic Republic of the Congo [15], Vietnam [16, 17], India [18], Philippines [19], Thailand [20], Indonesia [21], Lebanon [22] and other traditional societies, cultural norms and social unrest affect sexual relations, and maintaining virginity until marriage is common and mandatory so that the hymen is considered socio-culturally a sign of females’ chastity and righteousness [23].

Custom, culture and religion create an image of women who impose “female virginity” on their communities [15]. Attitudes toward sexual relationship and its moral aspects have changed dramatically in many parts of the world in recent decades [24, 25]. Iran, as a conservative society, has experienced substantial social and attitudinal changes over the past decades [16–22].

The modern Iran is a blend of tradition and modernity, but its dominant culture is still traditional and takes pride in female virginity [26]. According to the traditional and conservative Iranian culture, premarital sexual relations are forbidden [27], female virginity is a honor, and it is valuable and necessary for a girl to maintain and take care of her virginity. Any damage to a girl’s hymen prior to marriage would mean great catastrophe and can have dire consequences for her and her family [28]. Versus, the increasingly large young population of Iran and its great influence from international developments, especially in the age of communication, and the modernization of social relations have laid the foundation for converting tradition to modernity [29].

With the development of modernity and modernism in Iranian society, the status of marriage has changed and has increased the age of marriage or celibacy, delay in marriage has increased extramarital relationships and sexual desire disorders [30]. Farahani et al. (2018) carried out a study on university girls and showed that one of the most important considerations for girls and even boys in sexual relations is to preserve their virginity [31]. However, there is evidence that premarital sexual behavior is on the rise among Iranian youth, especially in large cities, but it is often hidden with unknown details [27, 32–36].

In Rahmani et al.’s study (2016), participants cited “custom” as the main reason for the lack of premarital sexual relations and considered physical virginity the only red line for Iranian girls. In fact, those girls who wanted to experience sexual relations said that they were inclined to have any type of sexual relationship, but on the condition that their hymens would remain intact. Although physical virginity can be a deterrent to sexual contact for a significant proportion of single girls, it may be a factor of further vulnerability for another proportion of girls who have non-vaginal sexual experience [36]. Therefore, the lack of a common and reasonable concept of virginity in relation to the type of sexual relation and the dominant perception of virginity loss only through vaginal intercourse lead to an increase in sexually transmitted diseases and HIV [37, 38].

In Iran, between the first years after the Islamic Revolution and the end of the Iran-Iraq war, the issue of “boy-girl relationships” was less of a “social issue” due to people’s strong adherence to socio-cultural values and involvement in the war. However, in the 1970s, they gradually developed relationships due to fundamental social changes and the emergence of a new young generation with different values and beliefs. A change of values has led to broader social changes [39]. Therefore, based on the socio-cultural developments in Iran, it seems necessary to study Iranian girls perspective about the concept of virginity.

The importance of virginity can be discussed from different perspectives. On the one hand, it is identified as an important factor in controlling sexually transmitted diseases and unwanted pregnancies among adolescents and youth as a strategy to control the negative consequences of premarital sex [37]. On the other hand, the importance of physical preservation of virginity or the hymen may be associated with an increased prevalence of unprotected non-vaginal sexual intercourse, which puts girls’ sexual health at risk [40]. Therefore, virginity is closely related to sexual health and fertility and this issue should be investigated in more depth with regard to sexual health and fertility.

There is a kind of inconsistency and contradiction in understanding the concept of virginity in the modern Iranian society. Although the cultural and social values of this society emphasize the preservation of virginity, influential factors such as modernity, education, social networks, global communication, influence from advanced Western societies, have obviously led to the emergence of a freer sexual attitude associated with less significance of virginity [41, 42]. Therefore, since Iranian society is a blend of tradition and modernity, it can be an excellent context for a study on the role of virginity in the girls’ sexual behavior development. By recognizing the girls’ attitudes and beliefs in this regard, we are hoping to provide strategies for designing and implementing sexual health education programs, because studies based on empirical and indigenous research will prove more effective in promoting women’s sexual health in societies with similar cultures.

## Study aim

The aim of the present study is development of strategies to promote Iranian girls’ sexuality with focus of the role of virginity in the construction of their sexuality.

The specific objectives of this study are to:

1. To determine the girls’ perspective about the importance and role of virginity in the construction of the sexuality and its related factors.
2. To compare the girls’ perspective born in the 1970s, 80s and 90s about the importance and role of virginity in the construction of the sexuality and its related factors.
3. To identify and analyze the girls’ interpretations and experiences and its related factors about the importance of virginity and its role in the construction of the sexuality.
4. To determine strategies to promote healthy sexuality among Iranian girls.

## Method

### Study design

**Fig. 1:**
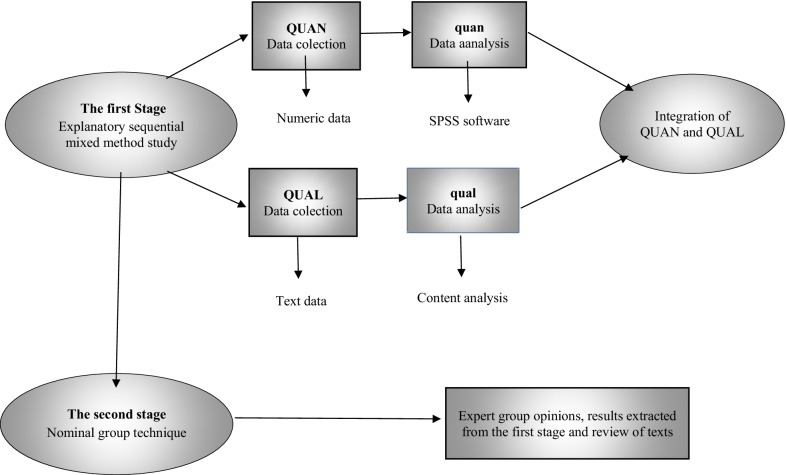
Study visual diagram

This is a two-stage study; an explanatory sequential mixed-methods follow-up design will be employed in the first stage, which consists of two quantitative and qualitative phases. First, a quantitative phase of the study will be performed, and after the quantitative data are analyzed, the qualitative phase of the study will be performed. In the end of the first stage, quantitative and qualitative data will be combined. In the second phase of this study, a nominal group meeting will be held with the participation of reproductive and sexual health experts (Expert Panel) to develop and present appropriate strategies to promote healthy sexuality in girls, so that the strategies extracted from the results of the first phase and review the texts in this meeting will be provided to experts and after receiving the opinions and ideas of the relevant group of experts in the meeting and prioritizing them, appropriate strategies to improve and promote the healthy sexuality of Iranian girls will be presented. The methodology of the steps is explained below (Fig. 1).

### Quantitative study

The quantitative phase of the study will be based on a cross-sectional population-based survey on the target population of Iranian girls living in Tabriz-Iran (the cohorts of the 1970s, 80s, and 90s).

### Sample size and sampling method

In order to estimate the sample size in the present research, the formula for the estimation of a proportion was employed.

$$ {\text{N}} = \frac{{\left( {\mathop z\nolimits_{1} - \frac{\alpha }{2}} \right)^{2} P\left( {1-P} \right)}}{{\mathop d\nolimits^{2} }} $$

In the present study, the z-value was equal to 1.9 at a 95% confidence level and the p-value based on the study of Honarvar et al. [32] in Iran and considering the d-value was equal to 10% around p (proponents of premarital sexual relationships), the sample size was obtained 459. Due to the use of cluster sampling in this study, the design effect was considered. In most cases, the numerical value of the design effect is considered to be about 1.5–2. It was considered 1.5 in this study and the final sample size was increased to 700 people.

$$ {\text{N}} = \frac{{\left( {1.96} \right)^{2} {0.456} \left( {1-0.456} \right)}} {{\left( {0.1 \times 0.456} \right)^{2} }} = 458.3 \cong 459 $$

$$ {\text{N}} = {459} \times {1}.{5} = {688} \cong {7}00 $$

Sampling in this study is based on population and cluster sampling method, because Tabriz is a large metropolis with different cultures. Attempts will be made in this study to give the whole population of the city a chance to be selected as a sample member. Due to social constraints and sensitivity of the issue of virginity and sexual intercourse and in order to ensure the participants’ privacy, the researchers will not interview the participants at their home. Therefore, the research location in this study will be considered health centers. The 1,559,000 population of Tabriz (according to the 2016 statistics) is distributed in ten urban districts. First, the health centers in Tabriz will be identified and a number of health centers will randomly be selected from each district (There are 83 health centers in Tabriz). The sample size will divided among the selected health centers based on quotas. Households that have a single daughter and meet the inclusion criteria will be randomly selected from among the health centers through the SIB website (This website covers more than 90–92 percent of the population of Tabriz, which is a desirable amount of coverage and the details of Iranian families with all its members are available in the SIB system in health centers and we can access these people). If a family has more than one unmarried girl, all of them will be included in the study if they wish. Then, they will be telephoned and those who are single, were born in the 1970s, 1980s and 1990s and agree to participate in the study, they will be asked how to participate in the study. If they agree and consent, an anonymous email or mobile number will be received from them and they will be sent a questionnaire link to fill out at home under stress-free conditions and in a confidential manner. Otherwise, they will be asked to attend the relevant health center on a specific date to be presented with the questionnaires to complete.

It should be noted that the cohorts of the 1970s, 1980s, and 1990s will be compared as a sub-goal in the quantitative phase of the study. Therefore, we mean the 1970s (born 1971.3.21 to 1981.3.20), the 80s (born 1981.3.21 to 1991.3.20) and the 90s (born 1991.3.21 to 2001.3.20).

### Inclusion criteria

Inclusion criteria in this study are: having Iranian nationality, no history of previous marriage or its contract as defined in the socio-cultural structure of Iran, having or lacking premarrital sex for girls, literacy, born in the 1970s, 1980s and 1990s and having no physical and mental illness as self-report.

### Exclusion criteria

Exclusion criteria are: unwillingness to continue participating in the study, and failure to complete the questionnaire.

### Scales and data collection

The data collection tool in the quantitative stage will be a researcher-made questionnaire to assess the girls’ views on virginity and its influential factors. This questionnaire has been designed after reviewing the relevant resources including studies by Carpenter (2002 and 2005) [4, 43], Barnett et al. [44], Eriksson et al. [45], Uecker et al. [46], Wilson et al. [47], Awwad et al. [22], Sprecher et al. [8], Landor et al. [1], Lipman et al. [48], Shirazi et al. [49], Hojat et al. [50], Motamedi et al. [42], Molla et al. [14], Arega et al. [51], Yaşan et al. [52], Teo et al. [20], Khalajabadi-Farahani et al. [31] and Rahmani et al. [53] and tools and based on the specialized experience of the research team. Since virginity and its influential factors are context-based concepts, the research team, having reviewed the existing sources, did not find a tool that socio-culturally suited investigation into these concepts. Therefore, a researcher-made questionnaire will be used to collect information in this phase of the study. The design of this questionnaire will be based on the conceptual framework of the research and its items will correspond to the theory of “social constructivism of sexuality”. This questionnaire contains four main parts:

*The first part* examines the participants’ demographic and socio-economic characteristics, including age, education, parental education, current life status, birth location, ethnicity, economic status, communication with parents, and the participants’ and their parents’ religious affiliation. The items in this part are 16 questions.

*The second part* is a questionnaire about the girls’ individual attitudes regarding the importance of virginity, with items such as “Girls should not have any premarital sexual intercourse”, “I believe that boys should remain virgins until marriage because of the importance of cultural and social beliefs”, “I preserve my virginity”, and “I abstain from any premarital sexual relations due to religious affiliation”. This questionnaire will be based on a 5-point Likert scale (totally agree, agree, no opinion, disagree, totally disagree). The items in this part are 12 questions.

*The third part* is a questionnaire about the girls’ interpersonal attitude questionnaire regarding the importance of virginity, with items such as “I preserve my virginity to respect my family values”, “Premarital preservation of virginity is very important for my female friends”, and “Boys deceive girls to be their sexual partners by claiming that all the girls have lost their virginity”. This questionnaire will be based on a 5-point Likert scale (totally agree, agree, no opinion, disagree, totally disagree). The items in this part are 12 questions.

*The fourth part* is a questionnaire about the girls’ cultural normative attitudes toward the importance of virginity with items such as “In our culture, virginity is particular to girls rather than boys”, “In our culture, a virgin bride is preferred”, and “Loss of virginity prior to marriage in our culture dishonors the girls”. This questionnaire will be based on a 5-point Likert scale (totally agree, agree, no opinion, disagree, totally disagree). The items in this part are seven questions.

The validity and reliability of these questionnaires will be measured before the commencement of sampling. The psychometric properties of the present researcher-made questionnaire will be examined before use in the following way:

*Face validity* In this study, face validity will be examined both qualitatively and quantitatively. In qualitative face validity, items will be examined in terms of level of difficulty, appropriateness and ambiguity. In quantitative face validity, a Likert scale will be considered for each item of the questionnaire and based on the responses of 10 members of the target group, the impact score of each item will be calculated.

*Content validity* In this study, content validity will also be examined both qualitatively and quantitatively. To determine the qualitative content validity, the researchers will consult 10 relevant experts regarding the content of the items, the general structure of the questionnaire and any removal or addition of the items (According to statistical tests available for instrument psychometrics, 10 experts are the minimum number in the panel of experts [54]). For quantitative content validity, they will use two indicators of CVR and CVI. In the content validity ratio, the necessity of an item inclusion will be evaluated from the point of view of the experts who respond each item with one of the following three options: (1) necessary (2) useful but unnecessary and (3) unnecessary. To calculate the content validity index, they will have 10 relevant experts evaluate the content validity using the three criteria of simplicity, clarity and specificity or relevance separately based on a 4-point Likert scale.

### Data analysis

The data will be analyzed using SPSS, 24. To describe the demographic and socio-economic characteristics, will be used descriptive statistics including frequency (percentage) and mean (SD) in the case of normal data distribution, and median (quartiles 25–75) in the case of non-normal data distribution. To compare girls’ perspective born in the 1970s, 1980s and 1990s about the importance and role of virginity in the construction of the sexuality, in bivariate analysis, one-way analysis of variance and chi-square tests will be used; in multivariate analysis, multivariate linear regression or multivariate logistic regression will be employed by controlling the demographic and socio-economic variables.

### Qualitative study

The data will be analyzed using the conventional content analysis approach [55]. Considering the objectives of the study in its qualitative stage, the use of this method can help the researcher to better understand the situation to explain the role of virginity in the construction of the sexuality among Iranian girls.

### Sampling method and data collection

Participants will be selected through purposive sampling. In the qualitative phase, they will be selected from among the girls who participate in the quantitative phase of the study and are willing to participate in the qualitative phase of the study, and based on either end of the response spectrum or the significance of the relevant factors of the sample. Attempts will be made to have maximum diversity in terms of underlying factors such as education, age, socio-economic status, religion and sexual relations. Interviews will also be conducted in places such as universities, health centers, or in places where participants feel more comfortable and safe. The respondents will be asked through semi-structured in-depth interviews, starting with the interview guide and an open-ended question, followed by questions about attitudes towards the importance of virginity and its role in the construction of the sexuality of Iranian girls to reach the qualitative research questions. The interview guide of the quantitative part contains the following questions:

- What individual factors have affected your perspective of virginity?
- To what extent has religious affiliation affected your perspective of virginity?
- To what extent has the family affected your perspective of virginity?
- To what extent have same- and opposite-sex friends and peers affected your perspective of virginity?
- To what extent has your society affected your perspective of virginity?
- What strategies do you suggest for healthy sexuality promotion in girls?

The research tool in the qualitative part is a researcher-made questionnaire. All interviews will be recorded by a digital tape recorder after the participants’ consent is obtained. The researcher will also take notes during the interview, recording the participants’ responses and facial expressions while they are expressing their specific experiences and views. The duration of the interview will be 45–60 min, which may vary somewhat depending on the experiences of individuals. Also, due to the sensitivity of the research topic and the shame of talking about sexual issues and premarital sex, it will be possible for participants not to tell the truth. The researcher will try to explain the importance of the research topic, establish communication and friendly interaction with the participants and observe the ethical principles in order to gain the trust and confidence of the participants and overcome this limitation.

### Data analysis

Immediately after data collection, they will be analyzed and the coding process began. Qualitative data analysis will be performed using conventional content analysis using the method attributed to Graneheim and Lundman.

Graneheim and Lundman explain, qualitative content analytical approaches focus on analyzing both the explicit or manifest content of a text as well as interpretations of the ‘latent content’ of texts that which can be interpreted or interpolated from the text, but is not explicitly stated in it [56]. Also conventional content analysis, coding categories are derived directly from the text data. With a directed approach, analysis starts with a theory or relevant research findings as guidance for initial codes. It should be noted that although the questions of the qualitative section are based on the theoretical framework, open codes will be used in data analysis and categories will be extracted from the data based on conceptual commonalities and in response to the research question. Therefore, the type of qualitative study approach will be of the conventional content analysis type [57].

### The second stage of research: nominal group technique

In the end of the first stage, quantitative and qualitative data will be combined for further explanation and interpretation to be used in the second stage to provide appropriate strategies for the girls’ healthy sexuality promotion.

The nominal group technique will be used to develop and present appropriate strategies to promote healthy sexuality in girls. The nominal group technique (NGT) is a structured group-based technique used to build consensus. Participants are asked to individually reflect and to generate ideas based on predetermined, structured questions asked by a facilitator [58].

To do so, first strategies will be extracted based on the results of the first phase as well as reviews of the literature, then a meeting will be held with reproductive and sexual health experts. Inclusion criteria for participants in the nominal technique include: reproductive and sexual health experts, residents of Iran, full acquaintance with Iranian culture and tradition, working in one of the centers related to girls’ health. Initially, the experts will be presented with the strategies extracted from the results of the first phase and the review of the texts, and any member of the group can comment on the main research question, and the ideas of the group members will be recorded. Then they are prioritized, and finally, appropriate strategies for improving and promoting the healthy sexuality of Iranian girls will be presented.

### Ethics approval and consent to participate

This study was approved by the ethics committee of the School of Nursing, Midwifery and Rehabilitation of Tehran University of Medical Sciences (ethics code: IR.TUMS.FNM.REC.1400.021). Inform written consent will be received from all participants in both quantitative and qualitative status. The participants will be assured about the confidentiality of information and privacy of their identity. It will also be explained that they are allowed to quit the study at any stage of intervention, and their refusal to cooperate is free at any stage, and no change will be made in presenting or in the quality of services offered to them.

## Discussion

Given that there is a different understanding of the concept of virginity in different parts of the world, so that it is considered a value in some societies but an antisocial behavior in others; it is particular to women and is governed by dual gender standards in some societies, while it is not gender-specific in others. All of these issues can be rooted in the religious, social, and cultural structures that dominate different societies [19, 59, 60].

Since the mentioned studies have different cultural, social and religious backgrounds from Iran where few studies have dealt with the concept of virginity only qualitatively [31, 59, 61, 62], it seems that the present study, by employing a better quantitative and qualitative approach, can clarify the young generation’s view in the socio-cultural background of Iran and explore the possible inconsistency and contradictions in the modern Iranian society to determine whether the traditional view of the concept of virginity in Iran is still dominant, or it has been changed by factors such as modernization, global communications, influence from advanced Western societies.

This study is one of the few studies that will be conducted in the field of sexual health and culture in Iran and will use a “mixed-methods” approach to determine and explain the role of virginity in the construction of sexuality from the Iranian girls perspective.

We hope that this study can present evidence-based documents from the latest physical, psychological and social developments in young Iranian girls’ sexuality and that the presented healthy sexuality promotion strategies, which will be based on Iranian socio-cultural developments, can provide the basic information required for policy-making and planning for young girls’ sexual health. It is also hoped that the findings of this study will be useful in culture-based sexuality education and support for reproductive and sexual health care for the young Iranian generation.

## Data Availability

Not applicable.

## Abbreviations

CVR: Content Validity Ratio
CVI: Content Validity Index
NGT: Nominal group technique
SD: Standard Deviation
